# Tribochemical Degradation at the Implant-Abutment Interface and Its Implications for Marginal Bone Loss and Peri-Implant Mucositis: A Narrative Review

**DOI:** 10.7759/cureus.115969

**Published:** 2026-09-08

**Authors:** Douglas J Silva Salas, Mariana Ocariz De Colina, Claudia Monteverde-La Roche, Diosvani Terrero-Reyes, Lucia Argüello-Lacayo, Karina Galvez-Armas, Luisana S Rodriguez

**Affiliations:** 1 Dentistry, University Jose Antonio Paez, Valencia, VEN; 2 Dentistry, Central University of Venezuela, Caracas, VEN; 3 Dentistry, University of Carabobo, Valencia, VEN; 4 Dentistry, University of Medical Sciences of Santiago De Cuba, Santiago De Cuba, CUB; 5 Dentistry, Catholic University of Honduras Our Lady Queen of Peace, Tegucigalpa, HND; 6 General Dentistry, Boston University School of Medicine, Boston, USA

**Keywords:** corrosion resistance, dental implants, implant-abutment interface, implant surface modification, marginal bone loss, passive film, peri-implantitis, peri-implant mucositis, titanium particles, tribocorrosion

## Abstract

The implant-abutment interface is the only non-osseointegrated junction of an implant-supported restoration and is continuously exposed to cyclic mechanical loading. Micromovement and fretting at this junction repeatedly disrupt the protective titanium dioxide film that confers corrosion resistance on titanium, so that mechanical wear and electrochemical corrosion act together in a process termed tribocorrosion. The resulting release of titanium ions and particulate debris into the peri-implant environment has been proposed as a contributor to peri-implant inflammation and marginal bone loss, yet its clinical significance remains uncertain. This narrative review synthesizes recent evidence on tribocorrosion at the implant-abutment interface, emphasizing repetitive passive-film disruption as the central mechanism of titanium degradation and the oral environment's modulating influence. The literature was retrieved from PubMed, prioritizing publications from the last five years and including narrative and systematic reviews, in vitro and finite element studies, and observational and clinical investigations addressing degradation mechanisms, biological consequences, peri-implant tissue changes, and preventive strategies. Experimental work consistently shows that connection geometry, preload maintenance, and material selection govern micromotion and microgap behavior, and that surface roughness influences bacterial adhesion and biofilm composition. Human studies confirm that titanium micro- and nanoparticles are commonly present in peri-implant soft tissues, including sites without a foreign-body reaction and without peri-implantitis, while particle uptake by macrophages is associated with oxidative stress, pro-inflammatory cytokine release, and increased receptor activator of nuclear factor kappa-B ligand signaling. Taken together, the available data support a bidirectional model in which tribocorrosion amplifies an existing inflammatory process rather than initiating it, and in which inflammation and instrumentation may in turn accelerate degradation. Tribochemical degradation may therefore contribute to peri-implant inflammation and marginal bone loss through mechanical instability, surface deterioration, and metallic debris release, but current evidence does not establish direct causality. Standardized tribocorrosion testing and longitudinal clinical studies are needed to clarify its clinical relevance and to guide preventive strategies.

## Introduction and background

Implant-supported rehabilitation is one of the most predictable treatments in contemporary dentistry, yet its long-term success depends on more than osseointegration alone. The implant-abutment interface is the only non-osseointegrated junction in an implant-supported restoration and is therefore its primary mechanical weak point. It transfers occlusal loads from the prosthetic restoration to the implant and surrounding bone while maintaining the assembly's structural stability, and it does so across an enormous number of loading cycles during the restoration's service life [1].

Because manufacturing tolerances and clinical procedures cannot eliminate microscopic discrepancies, a microgap of a few micrometers persists at this junction. Cyclic function generates micromovement and fretting within that gap, progressively reducing screw preload and altering the intimate fit between components [1,2]. Under these conditions, the protective titanium dioxide film that confers corrosion resistance on titanium is repeatedly disrupted and reformed. The coupled action of mechanical wear and electrochemical corrosion, known as tribocorrosion, has consequently been proposed as a mechanism of progressive surface degradation and the release of titanium ions and particulate debris into the peri-implant environment [3,4].

This proposal has clinical significance because titanium micro- and nanoparticles are now detected routinely in peri-implant soft tissues, in diseased sites and in clinically healthy ones alike [5,6]. Whether such degradation initiates peri-implant inflammation and marginal bone loss, merely accompanies them, or is amplified by them remains unresolved, and the distinction matters for how clinicians interpret metallic debris and which preventive measures are worth adopting.

This narrative review addresses the following central question: How does tribochemical degradation at the implant-abutment interface contribute to marginal bone loss and peri-implant inflammation through mechanical wear, corrosion, and metallic ion release? To answer it, the review examines the biomechanical behavior of the interface, the mechanisms of tribochemical degradation, the biological consequences of ion and particle release, the relationship between degradation and peri-implant disease, the strengths and limitations of the current evidence, and the biomaterial and bioengineering strategies now being developed to mitigate the problem.

## Review

We retrieved the literature from PubMed using combinations of the terms tribocorrosion, fretting corrosion, implant-abutment interface, titanium particles, titanium ions, peri-implantitis, peri-implant mucositis, and marginal bone loss. We prioritized publications from the last five years but retained earlier work when it established a mechanism that has not since been superseded. We considered narrative and systematic reviews, in vitro and finite element studies, and observational and clinical investigations addressing degradation mechanisms, biological consequences, peri-implant tissue alterations, and preventive strategies. Consistent with the narrative design, we selected studies for their contribution to the mechanistic argument rather than through a formal systematic protocol, and we did not attempt a quantitative synthesis. In total, 59 publications met these criteria and formed the evidence base for this review.

Biological and mechanical foundations of the implant-abutment interface

The success of implant-supported rehabilitation depends not only on predictable osseointegration but also on preserving the implant-abutment interface's biomechanical integrity. During mastication, swallowing, and parafunctional activity, this junction is subjected to complex axial, oblique, and lateral forces and undergoes countless loading cycles throughout the restoration's lifespan. Its mechanical behavior therefore decisively influences long-term outcomes, extending well beyond simple load transmission to the structural integrity of the entire implant-prosthetic complex [1].

To improve performance at this junction, implant-abutment connections have evolved from external hexagonal designs toward internal hexagonal, conical (Morse taper), locking taper, and hybrid configurations [1]. Although these designs differ geometrically, they share a common objective: to optimize mechanical engagement, preserve screw preload, improve stress distribution, minimize micromovement, and reduce prosthetic complications. They are best understood not as shape variations but as distinct biomechanical strategies for maintaining intimate contact between components under cyclic functional loading [7-9].

Current evidence consistently shows that connection geometry modulates the mechanical response of implant-supported restorations under cyclic loading. Systematic reviews indicate that all implant-abutment connections undergo a sustained reduction in preload during function, but that hexagonal connections tend to exhibit greater torque loss than internal conical and Morse taper systems, which maintain preload more effectively and provide greater joint stability [1,4]. Reviews of Morse taper and locking taper systems similarly report that improved mechanical engagement promotes more favorable stress distribution, greater reverse-torque stability, high long-term survival rates, and stable marginal bone levels, supporting the view that connection design strongly influences mechanical stability [8,9]. Randomized clinical evidence tempers this conclusion: when implant macrodesign and surface characteristics are standardized, external hexagonal, internal hexagonal, and conical connections yield comparable short-term biological and mechanical outcomes, indicating that connection geometry alone cannot fully explain long-term implant survival [7].

Mechanical integrity depends not only on connection geometry but also on preserving intimate adaptation between components. Because manufacturing tolerances and clinical procedures cannot completely eliminate microscopic discrepancies, a microgap inevitably exists at the implant-abutment junction [9,10]. Although these discontinuities measure only a few micrometers, they are biomechanically relevant because they modify load transmission, reduce contact stability, and alter the prosthetic assembly's mechanical behavior. Experimental work has also shown that clinicians have limited capacity to detect small implant-abutment misfits during routine evaluation, meaning that restorations judged clinically acceptable may still harbor microscopic discrepancies that affect long-term mechanical behavior [10,11].

Functional loading intensifies these challenges. During cyclic mastication, recurrent micromovements at the implant-abutment interface progressively reduce screw preload and modify component fit. As preload decreases, connection stability may be compromised, favoring microgap enlargement and increasing susceptibility to mechanical fatigue and screw loosening [1]. Three-dimensional analyses using micro-computed tomography have shown that thermodynamic aging significantly increases microgap volume in internal conical connections and that connection geometry exerts a greater influence on gap enlargement than the restorative material does [11]. Systematic comparison of titanium and zirconia abutments has likewise shown that zirconia components exhibit greater wear, more severe surface damage, and increased interface misfit after mechanical cycling, indicating that material properties contribute directly to the long-term mechanical integrity of the implant-abutment complex [12].

Taken together, these findings show that the implant-abutment interface should not be interpreted as a static, passive connection but as a dynamic biomechanical system conditioned by connection geometry, preload maintenance, cyclic fatigue, component adaptation, and material properties [13]. These factors interact continuously and, although they do not necessarily produce immediate clinical failure, they progressively alter the mechanical environment of the complex, promoting surface deterioration and loss of structural stability during long-term function [14].

Tribocorrosion: mechanisms of tribochemical degradation

Titanium is widely used in implantology because of its biocompatibility, mechanical strength, and corrosion resistance [13]. Its electrochemical stability depends on the spontaneous formation of a passive titanium dioxide film that acts as a protective barrier between the metal and the biological environment, and implant surface stability depends on the integrity of that film [4,14].

The oral environment, however, is dynamic and influences both surface behavior and material function [13]. Micromovements from repetitive occlusal loads, particularly at the implant-abutment interface, challenge the stability and maintenance of the passive layer [2] and together create conditions for tribocorrosive degradation [13].

During function, implants undergo cyclic loading. The implant-abutment interface is a critical zone in which masticatory forces, micromovements, and microgaps converge to generate fretting and sliding wear [2]. This mechanical action disrupts the passive oxide layer and exposes the underlying titanium to the corrosive environment, favoring electrochemical degradation [3,4,13-15]. Corrosion, in turn, alters the surface and promotes further wear, so the two processes reinforce one another and cause progressive material deterioration [3,4,14].

After mechanical disruption of the passive layer, the titanium surface can repassivate and partially restore its protective function [4]. The layer is therefore best regarded as a dynamic barrier whose stability depends on a continuous balance between disruption and reformation [3,14]. During tribocorrosion, repetitive motion between contacting surfaces repeatedly breaches the film, exposing fresh metal and promoting corrosion [3]. Recurrent mechanical disruption combined with slow or incomplete repassivation can therefore produce cumulative surface degradation. Degradation is not an isolated event but a repetitive sequence of disruption, depassivation, corrosion, and repassivation [3,4,13-15].

Although repassivation can occur rapidly, repetitive loading during function may prevent the oxide layer from fully stabilizing before it is disrupted again, and repeated cycles can lead to cumulative material loss and progressive degradation of the implant surface [2,13,14].

Tribocorrosion can accordingly be defined as a dynamic, synergistic process in which mechanical wear and electrochemical corrosion interact and mutually reinforce one another [3]. In titanium, this interaction involves repeated disruption of the passive film followed by depassivation and repassivation that, under continuous functional loading, contribute to progressive surface degradation [3,4,13-15].

The oral environment further modulates the process through interactions with saliva and its chemical and biological components [15]. In addition to minerals, saliva harbors microorganisms that form biofilms, alter local pH and chemistry, produce corrosive metabolites, and promote localized corrosion [4,15]. These local changes can interfere with passive-film stability and reformation, contributing to cumulative surface degradation [4,13-15]. High fluoride concentrations can also destabilize titanium dioxide by forming titanium-fluoride complexes, promoting degradation [16].

Tribocorrosion should therefore be understood as a dynamic process in which mechanical disruption of the passive film, electrochemical corrosion, and repassivation capacity interact under functional loading [3,4,13-15].

Metallic ion and particle release: biological consequences

Endosseous dental implants are composed principally of commercially pure titanium or titanium-based alloys, including titanium-aluminum-vanadium and titanium-zirconium alloys [17]. Their biocompatibility and corrosion resistance derive largely from the spontaneous formation of a titanium dioxide layer on the surface. Tribocorrosive processes at the implant-abutment interface can disrupt this protective layer, releasing metallic ions and particulate debris into the surrounding peri-implant environment [4].

Tribocorrosion products include titanium ions, titanium micro- and nanoparticles, alloying elements such as aluminum and vanadium, and mixed metallic particles. Their biological effects vary according to their physicochemical properties and the magnitude of local exposure [18]. Ionic and particulate degradation products elicit distinct responses: soluble metallic ions exert concentration-dependent cytotoxic and oxidative effects, whereas particulate debris is recognized and internalized by resident and infiltrating immune cells, particularly macrophages [4,19].

Particle dimensions and local concentration modulate the cellular response, and greater amounts of nanoscale titanium debris have been associated with increased cytotoxicity and inflammatory activity [18]. At the nanoscale, interactions with extracellular components, including proteins, may facilitate subsequent cellular uptake. Particulate material therefore appears to promote inflammation through mechanisms distinct from the concentration-dependent effects of soluble ions [4,18].

After cellular uptake, titanium particles can alter macrophage activity and promote a pro-inflammatory environment characterized by increased oxidative stress and reactive oxygen species generation [6]. These events activate inflammatory signaling pathways, including inflammasome-mediated responses, resulting in the production and release of pro-inflammatory mediators such as tumor necrosis factor alpha, interleukin 1 beta, and interleukin 6 [6,19]. Metallic debris may also influence the expression of the receptor activator of nuclear factor kappa-B ligand, linking the inflammatory response to altered bone remodeling [6].

Beyond macrophage-mediated inflammation, titanium degradation products affect several cell populations involved in peri-implant tissue homeostasis. Exposure to titanium debris can interfere with osteoblast function and osteogenic activity, while altered osteocyte signaling favors pathways associated with osteoclastogenesis and bone resorption [20,21]. These observations indicate that degradation products influence the cellular mechanisms that maintain peri-implant bone [20,21].

The resulting inflammatory microenvironment may further disrupt the balance between bone formation and resorption. Increased receptor activator of nuclear factor kappa-B ligand signaling favors osteoclast differentiation and activation, whereas oxidative stress and inflammatory mediators impair osteoblast function. Sustained exposure to titanium degradation products may therefore shift peri-implant bone remodeling toward resorption [20,21]. The biological consequences of tribocorrosion should thus be understood as the combined result of ion toxicity, particle-induced inflammation, and progressive changes at the implant surface and in the surrounding tissues [22].

Tribocorrosion and peri-implant disease: from mucositis to marginal bone loss

Peri-implant diseases arise from a multifaceted interaction between microbial biofilms, the host immune response, and local factors related to implant design and function [23]. Tribocorrosion has emerged as a potential contributor within this environment: mechanical and chemical wear modify implant surfaces and generate titanium-based particles that migrate into surrounding tissues. Although these alterations are well documented, their clinical relevance remains uncertain, and it is unclear whether surface degradation precedes inflammatory change or becomes more pronounced only after disease is established [6].

Surface characteristics influence early bacterial colonization. Experimental models show that both implant material composition and surface topography affect microbial adhesion. Choi et al. reported that *Streptococcus gordonii* formed denser biofilms on rough titanium surfaces and that *Fusobacterium nucleatum* and *Porphyromonas gingivalis* displayed distinct preferences depending on surface features [22]. Further studies have confirmed that roughness variations alter bacterial attachment patterns [23]. Clinical evidence nevertheless suggests that surface properties alone do not dictate disease progression: in a study comparing different abutment surfaces, Anitua et al. found that patient-related factors and time influenced biofilm composition more than surface treatment [24]. These findings support a multifactorial model in which surface deterioration may facilitate microbial retention without independently driving peri-implant disease.

Titanium debris has also been observed in tissues affected by peri-implantitis. Rakic et al. identified titanium particles in every granulation-tissue sample from peri-implantitis lesions in their case-control study [25]. Notably, these particles did not provoke a foreign-body reaction; instead, peri-implantitis tissues exhibited a more intense chronic inflammatory infiltrate, greater macrophage presence, and more neovascularization than periodontitis controls. Histological and immunohistochemical analyses have similarly documented inflammatory cell infiltration and alterations in the peri-implant bone microenvironment in affected patients [26]. While such observations show that titanium debris and inflammation frequently coexist, they do not establish a causal link between metal particles and tissue destruction [27].

Recent clinical studies have explored possible interactions between titanium release, the peri-implant microbiome, and host immunity. Casarin et al. reported distinct microbial and inflammatory profiles in peri-implantitis and found associations between local titanium levels, microbial community structure, specific pathogenic taxa, and elevated T helper 17 cytokines in peri-implant crevicular fluid [27]. These findings outline a plausible biological pathway connecting material degradation, dysbiosis, and inflammatory signaling that may contribute to tissue breakdown and impaired bone remodeling, although the cross-sectional design limits conclusions about the sequence of events [28,29].

The temporal relationship remains clinically relevant because titanium release may also occur after disease onset. In a randomized clinical trial, Daubert et al. observed titanium dissolution following nonsurgical instrumentation of implants with peri-implantitis, with more abrasive methods producing greater release [28]. A recent clinical report documented titanium particles in peri-implant tissues up to nine years after placement, predominantly within fibrous tissue and without evidence of marked inflammation [29]. These findings caution against interpreting metallic debris as inherently pathogenic [30].

Current evidence therefore supports a bidirectional model rather than a linear causal pathway. Tribocorrosion may alter implant surfaces, release metallic by-products, influence host-microbiota interactions, and contribute to the transition from mucositis to marginal bone loss; conversely, inflammation and clinical instrumentation may accelerate implant degradation [31]. On the available data, tribocorrosion is best understood as a contributing factor and disease amplifier rather than a proven independent initiator of peri-implantitis.

Current evidence: experimental models, clinical findings, and preventive strategies

Evidence on tribocorrosion at the implant-abutment interface derives predominantly from in vitro investigations, complemented by a smaller but increasingly relevant body of human tissue and clinical studies. Laboratory models permit controlled evaluation of mechanical wear and electrochemical degradation by combining cyclic loading, fretting or sliding conditions, and simulated oral electrolytes [30]. Electrochemical techniques quantify corrosion potential, current density, and passive-film stability, while scanning electron microscopy, energy-dispersive X-ray spectroscopy, and X-ray photoelectron spectroscopy characterize surface damage, elemental composition, and alterations in the protective oxide layer. Finite element analysis further identifies stress concentrations and micromotion at implant-abutment connections, although numerical predictions require experimental and clinical validation [30,31].

Experimental evidence consistently demonstrates that mechanical surface disruption and corrosion interact synergistically. A systematic review with meta-analysis showed that surface treatment significantly influences the tribocorrosion behavior of titanium implant alloys, supporting surface engineering as a potential preventive strategy [30]. An in vitro study combining fatigue testing, electrochemical analysis, and scanning electron microscopy showed that mechanically altered titanium implant surfaces changed corrosion resistance and mechanical behavior, emphasizing that procedures that disrupt the passive surface can modify material performance [31]. Such models are valuable for mechanistic investigation but cannot fully reproduce saliva composition, microbial biofilms, fluctuating pH, occlusal variability, or host inflammatory responses [32].

Human evidence confirms that metallic particles can be detected in peri-implant tissues, but their clinical significance remains contested. Olander et al. identified metallic particles using scanning electron microscopy with energy-dispersive X-ray spectroscopy and investigated inflammatory gene expression in mucosa adjacent to titanium and zirconia abutments, demonstrating the feasibility of linking material-derived debris to local biological responses [32]. Kheder et al. further demonstrated altered macrophage polarization, lymphocyte-related pathways, and immune signaling in peri-implant gingival tissues associated with titanium particles [33]. More recently, Dionigi et al. showed that titanium microparticles are commonly present in peri-implant soft tissues, particularly near the implant-tissue interface, and that particle density was not independently associated with peri-implantitis, underlining that particle detection should not be interpreted as evidence of causation [5].

This distinction matters clinically when evaluating marginal bone loss, inflammatory biomarkers, implant survival, and prosthetic complications. Tribocorrosion may contribute to an inflammatory microenvironment, but marginal bone remodeling and peri-implant disease are multifactorial and influenced by biofilm, implant position, prosthetic design, connection stability, host susceptibility, and mechanical loading [34]. Screw loosening can similarly increase micromotion and fretting at the interface and potentially accelerate wear, yet clinical studies directly linking screw loosening to titanium ion concentration, inflammatory biomarkers, and subsequent marginal bone loss remain scarce [35].

Preventive strategies should therefore target both mechanical stability and corrosion resistance. Stable internal or conical implant-abutment connections, adherence to manufacturer-recommended torque, preservation of screw preload, and control of unfavorable occlusal loading may all reduce micromotion and fretting. Surface modifications and protective coatings offer a complementary approach, since appropriately engineered surfaces improve corrosion and wear resistance [30]. Ceramic and zirconia components may reduce direct metal exposure in selected applications; however, contemporary randomized evidence indicates that zirconia should not automatically be considered biologically superior to titanium, with comparable short-term marginal bone and peri-implant outcomes reported for both materials [34]. Novel titanium alloys and corrosion-resistant coatings remain promising but require long-term clinical validation.

Overall, the current evidence provides strong mechanistic support for tribocorrosion and confirms the presence of metallic debris in human peri-implant tissues, but it has not established a direct causal pathway from implant-abutment degradation to mucositis, marginal bone loss, or implant failure [35]. Heterogeneous experimental protocols, small clinical samples, inconsistent particle-detection methods, and limited longitudinal data remain the principal limitations. Future studies should integrate standardized tribocorrosion testing with longitudinal measurement of metallic ions, inflammatory biomarkers, prosthetic complications, and radiographic bone changes to determine the true clinical relevance of implant-abutment degradation [36].

Future perspectives: advanced biomaterials and bioengineering approaches

Titanium-zirconium alloys are a promising alternative to conventional titanium alloys; their smaller grain size and increased surface oxide formation may improve corrosion resistance [35]. High-performance ceramics, particularly yttria-stabilized zirconia, have also emerged as potential alternatives to titanium, demonstrating favorable mechanical properties, biocompatibility, and osseointegration potential, along with a possible advantage in reduced plaque accumulation [34,36,37].

Diamond-like carbon coatings are promising protective treatments for titanium implant components. Their high hardness, low friction, and wear and corrosion resistance may reduce tribocorrosion, and such coatings have been shown to improve the biomechanical endurance and corrosion behavior of titanium implant abutments [38]. Graphene-based coatings offer another strategy, providing antibacterial, bioactive, and corrosion-protective properties while supporting osseointegration; silver-decorated graphene oxide and chitosan coatings applied to titanium dental implants have shown favorable biocompatibility and corrosion behavior in preliminary work [39,40].

Nano-engineering of dental implants aims to fabricate isotropic or anisotropic nanoscale features that stimulate cell bioactivity, promote implant integration, and improve implant physiological function. Nano-engineered surfaces can additionally confer bactericidal properties and enable localized drug delivery [41,42]. Modifying titanium with silver nanoparticles is a related strategy to mitigate infection risk and early implant failure [43], as are antibacterial coatings that release therapeutic agents from nanoparticle reservoirs anchored to the oxide surface [44].

Self-repair of the implant surface is, in one sense, an intrinsic property of titanium: the passive titanium dioxide layer repassivates spontaneously in physiological media, and this natural self-healing has been visualized electrochemically on titanium dental root implants [45]. Engineered self-healing coatings that actively repair microdamage and maintain the protective barrier extend this principle and could plausibly reduce corrosion and tribocorrosion, but so far have been developed mainly for other biomedical metallic substrates and remain largely experimental in dental implantology [46].

Antibacterial implant surfaces aim to reduce bacterial adhesion and biofilm formation, thereby decreasing peri-implant inflammation while preserving surface integrity. Surface modifications incorporating antimicrobial elements such as silver, zinc, magnesium, and copper have demonstrated antimicrobial activity in vivo; titanium-copper alloys, for example, exert a documented anti-infective effect associated with changes in the oral microbiota [47].

Corrosion-resistant nanocoatings may improve the electrochemical stability, biocompatibility, and longevity of titanium implants. Techniques including grit blasting, acid etching, and selective laser melting have been applied to titanium dental implants to reduce corrosion, improve osseointegration, and enhance biocompatibility [48,49]. Bioactive surfaces, including calcium phosphate coatings and biomolecule-based modifications such as crosslinked collagen type I, are designed to promote bone formation and improve osseointegration [50].

Artificial intelligence is a promising tool for evaluating implant prognosis, although further development is required. By integrating clinical and radiographic data, it may support treatment planning and predict implant outcomes [51]. Digital twins, defined as dynamic virtual representations of a physical system built from real-time data and computational modeling [52], could eventually provide patient-specific models for biomechanical simulation and prediction of implant behavior under functional loading; their translation into dentistry, however, is still emerging and has not yet been comprehensively mapped [53]. Designing dental prostheses with computer-aided design software is complex and time-consuming, so artificial intelligence has increasingly been integrated into the design process. Artificial intelligence-assisted personalized prosthetic design may improve occlusal morphology and force distribution, potentially reducing the mechanical overload that contributes to wear and degradation of the implant-restoration system [54].

Future research should focus on long-term clinical trials that can determine whether advanced materials and surface modifications genuinely reduce tribocorrosion, particle release, and peri-implant inflammation over time [55]. Standardized tribocorrosion testing is equally necessary to enable reliable comparisons between implant materials under clinically relevant mechanical, chemical, and biological conditions [56]. Managing established disease raises a related concern. Peri-implantitis often requires surgical intervention, and implantoplasty is used to arrest disease progression by mechanically removing the contaminated surface [57]. However, this procedure reduces fatigue and corrosion resistance and increases titanium particle release, so its mechanical and biological costs must be weighed against its therapeutic benefit [57,58]. Biomarker development, including salivary titanium monitoring, may offer a non-invasive way to detect implant degradation and peri-implant disease early and improve long-term outcomes [58]. Finally, personalized implant selection that integrates anatomy, bone quality, biomechanics, occlusal loading, implant material, and surface characteristics could optimize treatment for individual patients [59].

## Conclusions

Tribochemical degradation at the implant-abutment interface arises from a coherent chain of events: cyclic loading generates micromovement and fretting within an unavoidable microgap; this mechanical action repeatedly disrupts the passive titanium dioxide film, and the resulting synergy between wear and electrochemical corrosion releases titanium ions and particulate debris into the peri-implant environment. The biological consequences of that release are well characterized in vitro, where particulate titanium promotes macrophage-mediated inflammation, oxidative stress, and signaling that favors bone resorption over formation. Translating this mechanism into clinical causality is not yet justified, however, because titanium micro- and nanoparticles are commonly found in peri-implant soft tissues even in the absence of a foreign-body reaction and peri-implantitis; particle density has not been shown to be independently associated with disease; and titanium release also occurs after disease onset, notably following nonsurgical instrumentation and implantoplasty. The evidence therefore fits a bidirectional model in which tribocorrosion contributes to and amplifies peri-implant inflammation and marginal bone loss without being established as their independent initiator.

For clinical practice, this implies that prevention should target the mechanical origin of the process rather than the debris alone, through stable connection designs, correct torque and preload maintenance, control of unfavorable occlusal loading, and judicious selection of instrumentation and surface-modifying procedures. Emerging biomaterials, protective coatings, nano-engineered surfaces, and artificial intelligence-assisted planning are promising but remain unvalidated over clinically meaningful periods. Resolving the question posed by this review will require standardized tribocorrosion testing protocols together with longitudinal clinical studies that measure metallic ion and particle release, inflammatory biomarkers, prosthetic complications, and radiographic bone changes in the same patients over time; until such data exist, tribochemical degradation is best regarded as a plausible, mechanistically supported contributing factor to peri-implant disease rather than a proven cause.
